# Quality of anticoagulation management with warfarin among outpatients in a tertiary hospital in Addis Ababa, Ethiopia: a retrospective cross-sectional study

**DOI:** 10.1186/s12913-017-2330-0

**Published:** 2017-06-06

**Authors:** Teferi Gedif Fenta, Tamrat Assefa, Bekele Alemayehu

**Affiliations:** 1 School of Pharmacy, College of Health Sciences, Addis Ababa University, Addis Ababa, Ethiopia; 2 School of Medicine, College of Health Sciences, Addis Ababa University, Addis Ababa, Ethiopia

**Keywords:** Warfarin, Inr, Time in therapeutic range, Tikur Anbessa specialized hospital

## Abstract

**Background:**

Warfarin is the most widely used anticoagulant in the world. The difficulty of managing warfarin contributes to great potential for patient harm, both from excessive anticoagulation and insufficient anticoagulation. This study assessed the International Normalized Ratio (INR) control outcome measures and warfarin dose adjustment practices at cardiology and hematology outpatient clinics at a teaching hospital in Addis Ababa, Ethiopia.

**Methods:**

The study was based on a cross - sectional study design involving 360 retrospective patients’ chart review among outpatients who received warfarin for its various indications.

**Results:**

The mean frequency of INR monitoring per patient was 62.9 days (17.2–143.7 days).

Patients spent 52.2%, 29.0% and 18.8% of the time in sub-therapeutic, therapeutic and supra-therapeutic ranges, respectively. The daily warfarin dose was increased 50.9% and 36.9% and decreased in 52.8% and 60.9% of the time for occurrences of sub-therapeutic and supra-therapeutic INRs to achieve target ranges of 2.0–3.0 and 2.5–3.5, respectively.

**Conclusion:**

The quality of anticoagulation management with warfarin among outpatients in Tikur Anbessa Specialized Hospital was sub-optimal. This was reflected by low Time in Therapeutic Range (TTR), longer than recommended INR monitoring frequency, and minimal actions taken to adjust warfarin dose after occurrences of non-therapeutic INRs.

## Background

Since 1954, warfarin has been the most commonly prescribed oral anticoagulant for prevention and treatment of thromboembolic events mainly due to ease of its administration [1]. A fundamental step in quality care research in anticoagulation is establishing that anticoagulation does in fact prevent thromboembolism. During the last 50 years, the efficacy of warfarin in the prevention and treatment of thromboembolic diseases has been firmly established, especially for atrial fibrillation, venous thromboembolism, and valvular heart diseases [2].

A necessary first step was the development of a standardized test to measure the degree of anticoagulation. The prothrombin time was used to monitor anticoagulation but was plagued by a lack of standardization between laboratories. This was resolved through the development and widespread adoption of INR, which allows results from different laboratories to be comparable [3–5]. Having agreed on a way to measure anticoagulation, investigators were now able to achieve consensus regarding optimal INR ranges for various indications [6].

Despite its efficacy, warfarin is notoriously difficult to manage: its therapeutic window is narrow, it has significant interactions with diet and other medications, and its action is affected by comorbid conditions and other inherent patient characteristics. Therefore, it is a major patient safety goal to improve the quality of oral anticoagulation care and there is a need to institute quality measuring programs [7].

A quality measure of anticoagulation management is used to ascertain the degree to which a given system is successfully coordinating care to accomplish a particular therapeutic goal [7, 8]. Quality of warfarin management is assessed by the proportion of time the patient is maintained within therapeutic range since an increased Time in Therapeutic Range (TTR) is associated with a reduction of hemorrhage and thromboembolism [9]. Quality of warfarin therapy in long-term care was not studied yet in Ethiopian health facilities. Thus, this study was designed to assess the quality of anticoagulation management with warfarin among outpatients in Tikur Anbessa Specialized Hospital (TASH), a large teaching hospital in Addis Ababa. Knowledge of the quality of anticoagulation management with warfarin is important to identify the the most feasible and appropriate intervention to enhance anticoagulation services of TASH.

In the current study we aimed at assessing the INR control measures (proportion/number of INRs within therapeutic range, TTR and standard deviation (SD) of INR values) for warfarin therapy and investigating the quality of warfarin dose adjustment practice (warfarin prescribing) for outpatients in the cardiology and hematology clinics (CHCs) of TASH.

## Methods

### The study setting

The study was carried out at TASH, Addis Ababa, Ethiopia. It is the biggest university teaching hospital in the country and its services are divided as outpatient, inpatient and emergency departments. The hospital has currently about 2000 medical and non-medical staff. It has more than 500 beds [10]. Cardiology and hematology clinics (CHCs) of TASH give health care services for patients who need treatment and prevention of cardiovascular and hematological disorders.

### Study design

The study was based on cross-sectional study design in which retrospective patients’ charts review was employed as the data collection technique. The study population was adult patients who had received warfarin for its various indications after visiting the CHCs of TASH.

### Sample size determination and patient selection

The charts of all patients age 18 and above years old and who had received warfarin for at least 3 months and had been followed in CHCs of TASH from January, 2011 to January, 2012 were reviewed. These criteria were set because it is difficult to assess the quality of INR outcome measures in new patients due to instability of INR readings at this stage. Accordingly, 360 patients’ charts were included in this study.

Information about demographic and clinical characteristics of patients such as age, sex, comorbid conditions, indications of warfarin therapy, target INR ranges, INR values, frequency of INR monitoring (time interval until next scheduled test) and dose of warfarin patient take per day was gathered using a standardized data abstraction format. After collecting this information from chart, INR control outcome measures: proportion (number) of INRs within therapeutic range, TTR and standard deviation (SD) of INR value [11] were determined and compared with past studies’ findings to assess the quality of INR monitoring. The assessment of quality of warfarin prescribing was made based on how non-therapeutic INRs were managed; that is lowering, increasing or omitting dose of warfarin [12]. The time the patients spent in therapeutic range was calculated by the fraction of INR’s in the range for different target range of warfarin indications. The following method (formula) was used to determine TTR [9].

$$ \begin{array}{l}\mathbf{TTR}=\underset{\bar{\mkern6mu}}{\mathbf{The}\ \mathbf{number}\ \mathbf{of}\ \mathbf{INRs}\ \mathbf{within}\ \mathbf{the}\ \mathbf{target}\ \mathbf{range}\ \mathbf{for}\ \mathbf{all}\ \mathbf{patients}}\\ {}\kern2.16em \mathbf{Total}\ \mathbf{number}\ \mathbf{of}\ \mathbf{INRs}\ \mathbf{during}\ \mathbf{the}\ \mathbf{selected}\ \mathbf{interval}\ \mathbf{of}\ \mathbf{time}\end{array} $$

### Data quality assurance and analysis

Data from chart reviews were based on a standardized data abstraction format to maintain consistency. Data were entered into a computer using Epi Info version 3.5.3 and analyzed by SPSS version 16 and where appropriate, results were expressed as percentages, mean scores, and means ± standard deviation or ranges.

## Results

### The socio-demographic characteristics of outpatients

Among 360 outpatients who were attended CHCs of TASH and received warfarin during the period of January, 2011 to January, 2012; 233 (64.7%) were females and the mean age was 35.3 (SD = 12.8) years, with age range from 18 to 85 years. These patients were diagnosed with multiple diseases as co-morbid conditions (Table 1).

**Table 1:** Socio-demographic characteristics and comorbidities of outpatients who were on warfarin therapy at CHCs of TASH, 2012 (*N* = 360)

Item Description		N (%)
Sex	Male	127 (35.3)
	Female	233 (64.7)
Age	18–30	155 (43.1)
	31–45	140 (38.9)
	46–60	42 (11.7)
	Above 60	23 (6.3)
Comorbid conditions	Hypertension	37 (10.3)
	Congestive Heart Failure	154 (42.8)
	Asthma	6 (1.7)
	Hyper/Hypothyroidism	3 (0.8)
	Pulmonary Hypertension	62 (17.2)
	Others ^a^	60 (16.7)

^a^Others include anemia, osteoarthritis, neurologic disorders, depression, peptic ulcer disease, gasteroesophageal reflux disease, rheumatoid arthritis, infectious diseases, renal disorders, HIV/AIDS, ischemic heart disease, hemiparesis, congenital heart disease, cancer, etc

### The primary indications for warfarin

**Fig. 1:**
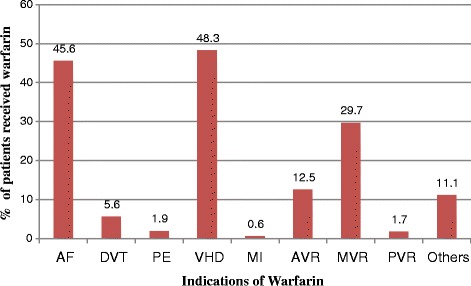
Indications of warfarin therapy for outpatients in CHUs of TASH, 2012

As shown in Fig. 1, the primary indications of warfarin therapy were valvular heart diseases (VHD), atrial fibrillation (AF) and mechanical mitral valve replacement (MVR). There were cases where patients received warfarin for more than one indication (48.9%). The INR target for atrial fibrillation, deep vein thrombosis, pulmonary embolism, valvular heart diseases, bioprosthetic valve replacement in mitral position antiphospholipid antibody syndrome, myocardial infarction, cardiac embolic stroke, cardiomyopathy, dilated cardiomyopathy and peripheral vascular disease was 2–3. Whereas INR target of 2.5–3.5 was used for mechanical valve replacement in mitral position, and for dual aortic and mitral mechanical valve replacement.

### INR distribution and outcome measures

**Fig. 2:**
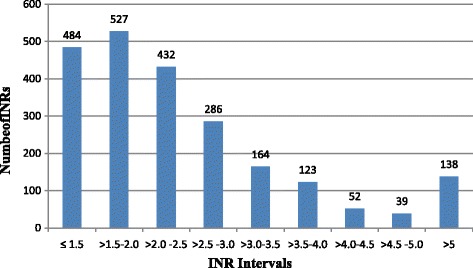
INR values distributions within different intervals for outpatients who were on warfarin at TASH, 2012

**Fig. 3:**
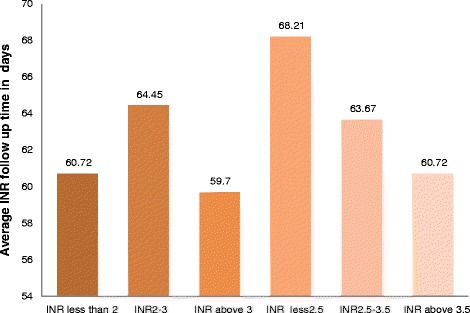
Average INR follow up time in days for different INR levels among outpatients who were on warfarin at TASH, 2012

**Fig. 4:**
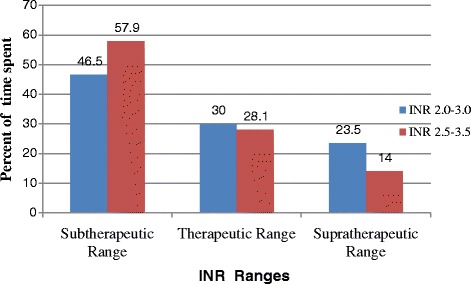
Time spent in sub-therapeutic, therapeutic and supra-therapeutic INR ranges for outpatients who were on warfarin at TASH, 2012

A total of 2245 INRs values were recorded over 12 months. They were divided into different categories to show how INRs were distributed. As indicated in Fig. 2, 484(21.6%) and 527 (23.5%) of the INRs values were ≤1.5 and in the range of >1.5 to 2, respectively. Out of all INR values, 138(6.1%) were above 5. The mean frequency of INR monitoring per patient was every62.9 days (range 17.7–143.7 days) irrespective of patients’ INR targets. Furthermore, average follow up time for INR values was calculated for both targets with their respective non-therapeutic INR monitoring frequency as described in Fig. 3. INR goal was 2.0–3.0 in 66.7% and 2.5–3.5 in 33.3% of patients. The mean INR reading per patient was 2.5 ± 0.8 and 2.6 ± 0.80 in those who should achieve INR of 2.0–3.0 and 2.5–3.5, respectively. The time patients spent in the sub-therapeutic, within therapeutic and supra-therapeutic ranges was shown in Fig. 4 for both INR rages. On average patients spent 52.2%, 29% and 18.8% of the time in sub-therapeutic, therapeutic and supra-therapeutic ranges, respectively.

### Warfarin dose adjustment practices at TASH

The assessment of quality of warfarin prescribing (daily warfarin dose adjustment practice) was made based on how non-therapeutic INRs were managed; that is lowering, increasing or omitting dose of warfarin [12]. In the target range of 2.0–3.0, for INRs that were <2.0, the average daily dose of warfarin for the days that followed was increased in 276 (50.9%) of such cases. In 190 (35.1%) of such cases, physicians did not change the previous dose of warfarin. However, for 76 (14.2%) of sub-therapeutic INRs of target range 2.0–3.0, the average daily dose of warfarin was decreased until next patient appointment date. Among 320 INRs which were above target range (>3.0), warfarin daily dose was decreased in 169 (52.8%) of occasions. But in 106 (33.1%) and 45 (14.1%) of occurrences (INRs > 3), warfarin daily dose was remained unchanged and increased for the days that followed, respectively (Table 2).

**Table 2:** Non-therapeutic INRs monitoring and daily warfarin dose adjustment in the CHUs of TASH, 2012

Target INR Range	Number of Non-therapeutic INRs	Decreased N (%)	Unchanged N (%)	Increased N (%)
Target Range 1	INR < 2 (*N* = 542)	76 (14.2)	190 (35.1)	276 (50.9)
	INR > 3 (*N* = 320)	169 (52.8)	106 (33.1)	45 (14.1)
Target Range 2	INR < 2.5(*N* = 331)	56 (16.9)	153 (46.2)	122 (36.9)
	INR > 3.5(*N* = 81)	49 (60.5)	17 (21)	15 (18.5)

*Target Range 1 = INR 2.0–3.0 Target Range 2 = INR 2.5–3.5*

For patients who were supposed to achieve a target range 2 (2.5 to 3.5), but were in sub-therapeutic range (INR < 2.5), physicians increased daily warfarin dose in 122 (36.9%), decreased in 56 (16.9%), and remained unchanged in 153 (46.2%) of cases. For the total of 81 INRs that were above the goal range (INR > 3.5), warfarin daily dose was decreased in 49 (60.5%) of the cases till the patients’ next visit.

## Discussion

This study explored the quality of anticoagulation management with warfarin in the CHCs of TASH. Quality of INR monitoring by physicians was unsatisfactory; as revealed by low TTR (29%) when compared with a goal of maintaining a therapeutic INR ≥ 50% of the time (the minimum threshold required to achieve a benefit from warfarin therapy) [12]. The potential reasons for low TTR might be prolonged duration of INR monitoring, longer appointment date due to patient load, absensce of a standard protocol for warfarin therapy management and lack of a anticoagulation clinic at the studied hospital.

The TTR obtained in the present study was also lower than the results documented in other studies [13–15]. A research done in the city of St. John’s, Canada among patients at family medicine clinic reported TTR of 65%, just over twice the present study result [16]. However, our result was similar to findings from a study in US hospital which reported 30.6% TTR [17], but higher than the result of a study done in India [18]. The low TTR might be due to less frequent monitoring (1INR within 63 days). According to one previous study, TTR can be increased with more frequent INR monitoring [18]. It has been also demonstrated that 50–60% of patients can be expected to remain in their target range if monitoring of INR occurs monthly, 77–85% if monitored weekly and up to 92% if monitored every 3 days [19]. Moreover, the present study revealed that patients spent 18.8% of the time in supra-therapeutic range. This figure was higher than results previously reported (11–14%) [12, 13, 15, 20].

The mean interval between two INR tests per patient was nearly 63 days among follow up patients in the CHCs of TASH which was very long even for patients who were receiving a stable dose warfarin. A study done in United State of America showed INR was monitored every 46 days [17]. Whereas, another study found that the mean number of days between two INRs determinations was 6.2 [11]. The longer INR monitoring frequency could be one of the main reasons for the lower TTR identified in this study. The America College of Chest Physicians and American Heart Association and other studies suggest that INR should be monitored at an interval of no longer than every 4 weeks for stable patients receiving warfarin [5, 8, 21].

Management of non-therapeutic INRs (quality of warfarin prescribing) by physicians was sub-optimal. In this regard, it was in only 276 (50.9%) and 122 (36.9%) of cases in which daily doses of warfarin increased for the days followed in response to occurrences of sub-therapeutic ranges in relation to target ranges of 2.0–3.0 and 2.5–3.5, respectively. The finding of the present study was very small when compared with what was found by Aspinall et al., [12] where in 75% of cases warfarin doses were increased following occurrences of sub-therapeutic ranges. Moreover, the present study indicated that warfarin dose adjustment at TASH was also minimal when INRs were in supra-therapeutic ranges. This was reflected by decreased daily dose of warfarin in only 169 (52.8%) and 49 (60.5%) of the cases in the two target INR ranges.

INR should be monitored within a week after the occurrences of non-therapeutic ranges to adjust daily warfarin dose accordingly [8]. Taking other alternative actions such as recommending non- pharmacological actions and managing warfarin interacting medications could be the reasons for providers’ poor response on warfarin dose adjustment for patients in non-therapeutic INR ranges [8, 22, 23]. However, our study didn’t assess the influence of the above alternative interventions due to poor patients’ medical charts recording practice in the units and high turnover of physicians in managing the same patient.

The anticoagulation services are being provided once in a week in TASH. This would influence the management of non-therapeutic INRs and warfarin dose adjustment. For managing the occurrence of INR readings out of the therapeutic ranges, both units should use warfarin dosing algorithms. High quality dose management is needed to achieve and maintain the INRs in the therapeutic ranges. Paper-based and/or computerized software models of warfarin-dosing protocols, use of point of care testing, anticoagulation management services with dedicated personnel (i.e. anticoagulation clinics) are recommended tools to provide a systematic approach to improve anticoagulation management services [17].

In settings like the CHCs of TASH where physicians are over burdenend with diagnosing and treating all patients’ disease conditions and a shortage of physicians is evident, clinical pharmacist can provide intense education to patients and their caregivers, check patients’ adherence more thoroughly, give extra attention to potential warfarin- drug/herbal supplement interaction at each clinic visit and can counsel patients who have poor adherence to their treatment and difficulty in INR control [24]. Pharmacist managed anticoagulation clinics are becoming common in hospitals of most countries and have shown improved patient outcomes including reduced hospital admissions for preventable embolism, bleeding, or treatment of thrombosis [16, 25–27]. For example, implementation of a pharmacist managed ambulatory care anticoagulation clinic in South Korea resulted in an increase in percentage of INRs maintained within therapeutic range from 66% in the usual care group to 82% with the new initiative [28]. Thus, based on finding from this study and internationational experiences, there are opportunities to start Pharmacist Managed Anticougulation Clinic in this biggest teaching hospital (TASH) in the country.

The present study is not without limitations. While auditing patients’ charts retrospectively, we faced a continuous challenge to extract the necessary information. This was mainly due to poor organization in documenting of patients’ history chronologically and; also illegible physician handwriting. Beside this, while searching charts of the study population from the hospital medical record room, we couldn’t find a large number of charts which might have important information due to poor documentation and arrangement. Moreover, the incompleteness of the patient charts hindered the ability to identify the number of bleeding or thromboembolic events from the study population.

## Conclusions

From this study it can be concluded that the quality of anticoagulation management with warfarin among outpatients who received warfarin was suboptimal. This was reflected by low TTR, longer INR monitoring frequency than is normally recommended, and minimal actions taken by physician to adjust daily warfarin dose after occurrence of non-therapeutic INRs.

## Abbreviations

CHCs: Cardiac and Hematology Clinics
INR: International Normalized Ratio
PMAC: Pharmacist Managed Anticougualtion Clinic
SD: Standard Deviation
TASH: Tikur Anbessa Specialized Hospital
TTR: Time in Therapeutic Range
